# An Image-Based Identification of Aggressive Breast Cancer Circulating Tumor Cell Subtypes

**DOI:** 10.3390/cancers15102669

**Published:** 2023-05-09

**Authors:** Mohamed Kamal, Yiru Jess Wang, Sarai Plummer, Amber Dickerson, Min Yu

**Affiliations:** 1 Department of Stem Cell Biology and Regenerative Medicine, Keck School of Medicine, University of Southern California, Los Angeles, CA 90033, USA; 2 USC Norris Comprehensive Cancer Center, Keck School of Medicine, University of Southern California, Los Angeles, CA 90033, USA; 3 Department of Zoology, Faculty of Science, University of Benha, Benha 13518, Egypt; 4 Department of Pharmacology, University of Maryland School of Medicine, Baltimore, MD 21201, USA; 5 Marlene and Stewart Greenebaum NCI Comprehensive Cancer Center, University of Maryland School of Medicine, Baltimore, MD 21201, USA

**Keywords:** breast cancer, circulating tumor cells, heterogeneity, morphometric analyses, prognosis

## Abstract

**Simple Summary:**

The majority of cancer-related deaths are attributed to distant metastases—the spread of cancer to different parts of the body. Accurate and early prediction of metastasis and response to therapy is key for the better management of cancer. Although metastases were initiated by circulating tumor cells (CTCs) shed into the bloodstream, only a small subset of CTCs bears metastatic capacity. Since cellular morphology has been shown to reflect cell state and function, we aim to detect the metastasis-initiating subpopulation of CTCs through a simple image analysis. We performed deep morphometric analyses of CTCs and determined their metastatic potential in mice. We identified a subgroup of CTCs with small size, large mitochondria and rough membrane texture to have the highest tumorigenic potential. Our new findings provide a simple image-based identification of CTC subpopulations with elevated aggressiveness, which is expected to provide a more accurate prediction of breast cancer patient survival than total CTC numbers.

**Abstract:**

Using previously established CTC lines from breast cancer patients, we identified different morphometric subgroups of CTCs with one of them having the highest tumorigenic potential in vivo despite the slowest cell proliferation in vitro. This subgroup represents 32% of all cells and contains cells with small cell volume, large nucleus to cell, dense nuclear areas to the nucleus, mitochondria to cell volume ratios and rough texture of cell membrane and termed “Small cell, Large mitochondria, Rough membrane” (SLR). RNA-seq analyses showed that the SLR group is enriched in pathways and cellular processes related to DNA replication, DNA repair and metabolism. SLR upregulated genes are associated with poor survival in patients with ER+ breast cancer based on the KM Plotter database. The high tumorigenic potential, slow proliferation, and enriched DNA replication/repair pathways suggest that the SLR subtype is associated with stemness properties. Our new findings provide a simple image-based identification of CTC subpopulations with elevated aggressiveness, which is expected to provide a more accurate prediction of patient survival and therapy response than total CTC numbers. The detection of morphometric and transcriptomic profiles related to the SLR subgroup of CTCs also opens opportunities for potential targeted cancer treatment.

## 1. Introduction

Breast cancer is the most prevalent cancer among women globally and remains a leading cause of death, despite advances in therapies [1]. The high mortality rate is mainly attributed to distant metastases. To address this, accurate and reliable disease monitoring is needed to detect metastasis early and allow for intervention. Current methods for monitoring metastasis such as imaging and tumor markers lack sensitivity and specificity. Therefore, there is still a need to overcome this unmet challenge.

Metastasis is a multistep process that starts when cancer cells shed from a primary tumor site and enter the bloodstream, making systemic circulation the key checkpoint between the primary and the secondary tumor sites [2]. These shed cells are known as circulating tumor cells (CTCs) and are considered a valuable source for liquid biopsy, as they contain the seeds of metastasis and enable longitudinal and minimally invasive disease monitoring [3,4,5,6]. 

Many clinical studies have obtained a significant amount of evidence for the clinical importance of CTC enumeration. In a landmark large multi-center prospective study, breast cancer patients with CTCs > 5 per 7.5 mL of blood have been shown to have a shortened progression-free survival (PFS) and overall survival (OS) compared to those who have <5 CTCs per 7.5 mL blood [3]. Similar adverse associations with prognosis were found in prostate [7] and colorectal [8,9] cancers and led to the subsequent FDA approval for usage in these cancers. These findings were further confirmed in several meta-analyses [10,11] and clinical trials [12,13,14,15,16] in metastatic breast cancers. In addition, metastatic breast cancer patients who have bone and visceral metastasis have been shown to have more CTCs than patients who have either bone or visceral metastasis, suggesting that multiple sources of tumors shed CTCs [17]. Furthermore, evidence also showed the value of CTC enumeration in treatment responses in metastatic breast cancers [10,11,14,17,18,19]. 

Beyond enumeration, the characterization of CTCs is mostly in its experimental observation phases but has led to the discovery of its extreme heterogeneity. For example, CTCs isolated from metastatic breast cancer patients have been shown to have a dynamic composition of their epithelial or mesenchymal states, which is associated with responses to treatment [20]. Discordance of the ER or HER2 status between the primary tumor and CTCs has been reported [21,22,23,24]. Several single-cell RNA-seq analyses of CTCs have shown extreme heterogeneity in their transcriptional landscape [25,26]. These findings are in accordance with the expectation that CTCs may reflect the temporal evolution of tumor cell subclones, during disease progression or therapy resistance [14,27], and only a subset of them have the ability to initiate metastasis. Indeed, a study has shown that the number of specific CTCs bearing either cancer stem cell or mesenchymal properties or both hold greater biological significance and impact on disease progression and clinical outcomes compared to the number of total CTCs [28,29]. Thus, resolving CTC heterogeneity may further enhance their potential as a cancer biomarker.

Here, we chose to investigate the cellular morphological heterogeneity of CTC subclones and their link to metastatic potential and molecular signature. Cellular morphology is a signature of a single cell state, matched to its function [30]. Hence, morphological heterogeneity of CTCs may reflect variations in their metastatic potential and serves as a fast and direct indicator of disease progression. Studies have established that the abnormal cytomorphology of CTCs in metastatic breast, colorectal, and prostate cancers is associated with poor clinical outcomes [31]. Understanding the morphometric subclasses of CTCs and their corresponding metastatic potential can deepen our knowledge of metastasis-initiating CTC properties. 

Studying CTCs composes of two challenges. First, CTC is a very rare population. Fresh isolated CTCs from patient samples are not enough for quantitative pipeline studies. Second, CTCs are very fragile. Certain isolation methods can alter the cell, making it not ideal for functional studies. Our lab overcame these challenges by using previously established six unstained CTC lines via ex vivo culture of CTCs isolated from breast cancer patients [32] and demonstrated that they recapitulate the major metastatic patterns in mice, as seen in the corresponding patients [33], ideal for downstream analysis. These lines provided us with sufficient materials to intensively investigate CTCs’ morphological heterogeneity. Here, using two CTC lines, we developed a workflow of single-cell 3D imaging, morphological feature extraction, high-content imaging clustering and RNA seq analyses, resulting in a deeper understanding of the morphological subclasses with higher tumorigenic capacity and associated transcriptional differences. 

## 2. Materials and Methods

### 2.1. Cell Culture and Cell Proliferation

We have previously established 6 breast cancer patient-derived CTC lines [32]. In this study, we used the CTC lines BRx68 and BRx07 for all immunofluorescence (IF) staining. Cells were cultured in RPMI 1640 media (Gibco, Waltham, MA, USA) supplemented with EGF (20 ng/mL) (Peprotech, Cranbury, NJ, USA), basic FGF (20 ng/mL) (Peprotech), B27 (10 mL) (Gibco), and antibiotic-antimycotic (Life Technologies, Carlsbad, CA, USA), in 6 well ultralow attachment tissue culture plates (Corning, Corning, NY, USA) at 37 °C, 5% CO_2_ and 4% O_2_. For monitoring of cell proliferation and morphometric characteristics of different CTC clusters, 1000 cells of each CTC cluster (sorted by FACS) were cultured in CTC media and the media was replaced every three to four days. Cells were counted under a phase contrast microscope and 10 to 20 individual cells from each CTC cluster were imaged using the automated fluorescence microscope BZ-X800 (Keyence, Itasca, IL, USA) every two weeks, as described below. At each time point, the total cell number was calculated, and a small volume of cell suspension was processed for IF staining and imaging. To calculate the proliferation rate, the total cell number at each time point was divided by that at the previous time point after deducting cells used for imaging.

### 2.2. Immunofluorescence Staining and Image Acquisition

BRx68 and BRx07 cells were fluorescently labeled live with 5 μM of the cytoplasmic live-cell dye CellTracker Green (ThermoFisher Scientific, Waltham, MA, USA), 20 μM of the nuclear dye Hoechst 33342 (ThermoFisher Scientific) and 1 μM of the mitochondrial dye Mito-Tracker DeepRed (Cell signaling, Danvers, MA, USA). Cells were incubated at 37 °C for 30 min, then washed three times with a staining buffer (phosphate-buffered saline (PBS) pH 7.2, 0.5% bovine serum albumin (BSA) and 2 mM EDTA). Cells were plated in CyteSlides (a chamber slide with a coverslip glass bottom) (RareCyte, Seattle, WA, USA) in the presence of a staining buffer. Slides were kept in the fridge at 4 °C for at least 1 h for cells to set to the glass bottom before imaging.

After staining, individual cells (one cell per frame) were imaged using the automated fluorescence microscope BZ-X800 (Keyence, Itasca, IL, USA) at 40× magnification. For each of the three-fluorescence channels, serial optical images were captured using the z-stack function of Keyence yielding an average of 20 images per channel with each optical slice of 0.9 μm thickness. BZ-X800 Keyence provides high-quality z-stacked images using its sectioning function which uses the brightest spot in each optical slice as a guide to removing background noise and fluorescence blurring yielding high-resolution confocal-quality optical sections. Exposure times of all channels were determined manually based on the level of oversaturation as detected by the sectioning function of Keyence and kept under 1 s.

### 2.3. Feature Extraction

Using ImagJ (release 1.53 m 28) [34], single images from respective fluorescence channels were stacked into 3D images, split into their different color components and only the frame with the relevant color was saved. CellProfiler 4.1.3 [35] based pipeline was developed for feature extraction from single-colored 3D images. Images from different fluorescence channels were segmented independently and their descriptors were extracted. The most centered section of each cell was used for 2D image analysis.

Segmentation of the CellTracker Green images was used as a guide for the cytoplasm and the outer border of the cell. A closing module was applied to close holes in the segmented CellTracker Green images. Closed images were then used for the extraction of the whole cell’s morphometric features, termed “Cell”, whereas segmented Hoechst 33342 and Mito-Tracker DeepRed images were used (without closing holes) for feature extraction of dense areas of the nucleus (termed “DenseNuc”), and mitochondria (termed “Mito”), respectively. Holes in the segmented Hoechst 33342 images were then closed using the closing module and used to extract features of the whole nucleus, termed “Nucleus”. 

For each of the four components, surface area, volumes, granularity and texture parameters were extracted. Granularity and texture were measured at multiple scales, in various directions and using different algorithms. Moreover, volumes and surface area ratios of Nuc/Cell, DensNuc/Cell and Mito/Cell were calculated, yielding a total of around 500 morphometric features per cell. A total of 500 cells were processed.

### 2.4. Image Data Analysis

We developed an R-based pipeline to analyze imaging data and detect different CTC morphometric subgroups and their unique morphological characteristics. First, quality control filters were applied where cells with no data across all features or features with no data across all cells were removed. Second, the remaining missing values were replaced with the mean of the feature. Third, data were log-transformed and a batch effect was detected. The batch effect was removed by normalizing data of each feature relative to each staining batch using the “Scale” function in R. Lower and upper outliers of each feature were detected as values which are lower or greater than the first or the third quartile, respectively, plus the interquartile range (IQR) multiplied by 3 as described in the equations below.

Lower outlier = First Quartile + IQR × 3

Upper outlier = Third Quartile + IQR × 3

Lower outliers were replaced by the first quartile value, whereas upper quartiles were replaced by the third quartile value. Dimensionality reduction and clustering analysis were performed using PCATools [36] and Pheatmap, respectively. Identification of unique morphometric characteristics of each group was performed using the Factoextra package, as follows. First, the optimal number of groups was determined using the “fviz_nbclus” tool. Second, cells were split into groups using the “CutTree” tool based on the optimal number of groups. Third, morphometric features were compared across groups using the “Plot” function.

### 2.5. Fluorescent Assisted Cell Sorting (FACS)

BRx68 cells were stained live with 3µM CellTracker Green and 500 nM Mito-Tracker DeepRed, as described above. Flow cytometry was performed using a BD FACSAria I cell sorter. Cell viability was determined by positive staining with CellTracker Green. The forward-scatter area by forward-scatter height (FSC-A~FSC-H) was used to discriminate single cells from doublets. Gating on the forward-scatter area by the side-scatter area (FSC-A~SSC-A) was used to distinguish small cells from large cells. We used a strict gating strategy for cells sorted for ScRNA-seq, where only the smallest 10% of cells were considered “Small” and the largest 10% of cells were considered “Large”. Small cells were further sorted based on Mito-Tracker DeepRed into “Small/High” and “Small/Low”. Small/High are small cells from the top 10% Mito-Tracker DeepRed positive cells (SLR), whereas “Small/Low” are small cells from the lowest 40% Mito-Tracker DeepRed stained cells (SSR). Sorted cells were investigated under the microscope to confirm the accuracy of sorting and ensure it matches the image analysis.

### 2.6. Single-Cell RNA-Sequencing Workflow

Single cells representing different CTC subgroups were sorted directly into 96-well plates (VWR) containing staining buffer. Plates were kept on ice and checked under a microscope for the presence of cells. Contents of wells that are confirmed to have single cells were transferred to PCR tubes. Cells were then processed using SMART Seq v4 Ultra Low Input RNA Kit for Sequencing (Takara, San Jose, CA, USA) according to the manufacturer’s instructions to generate single-cell cDNA libraries for mRNA sequencing. All cDNA samples were run on a TapeStation system (High Sensitivity D5000 DNA Analysis Kit as per manufacturer’s protocol). cDNA libraries were prepared using the Nextera XT DNA Library Prep Kit (Illumina, San Diego, CA, USA) with Nextera index kit index 1 (i7) and index 2 (i5) adapters. Libraries were sequenced on a NovaSeq6000 (Illumina) to obtain 150 bp-long paired-end reads. For each cell, read 1 and read 2 were merged into a single fastq file and processed as single-end reads.

RNA-sequencing reads were trimmed for Nextera and Illumina adapter sequences using Trim Galore under default parameters. Trimmed reads were then mapped to the human genome build GRCh38 from Ensemble (ftp://ftp.ensembl.org/pub/grch38/current/fasta/homo_sapiens/dna/Homo_sapiens.GRCh38.dna_sm.primary_assembly.fa.gz (accessed on 15 March 2022)) using STAR under optimized parameters for single-end sequenced data. Aligned reads were then counted via featureCounts [37] and piped into DESeq2 [38] for normalization to sequencing depth and downstream analysis. To produce the PCA plot, count data was transformed via the vst function to eliminate the experiment-wide trend of variance over mean and the plot was produced using ggplot2. The contrast function was used to compare different groups of samples under the FACS categories “SLR” vs. “SSR” and Small (SSR and SLR combined) vs. Large (LSS). For the SLR vs. SSR comparison, differentially expressed genes (DEGs) with a False Discovery Rate (FDR) of 0.05 and log2 fold change of greater than 2 or less than −2 were detected. Clustering analysis, supervised by DEGs, was performed using the ComplexHeatmap [39] package to test their ability to differentiate between different CTC subgroups. For both comparisons, up-regulated genes with an FDR of 0.05 and log2 fold change of greater than 2 were piped into EnRichr [40] to predict the enrichment of signaling pathways, and functional and cellular states.

### 2.7. In Vivo Tumorgenicity Assay

Tumorgenicity of different CTC morphometric subgroups was tested in vivo using mammary fat pad (MFP) implantation. All animal experiments were carried out in accordance with approved protocols from the Institutional Animal Care and Use Committee of USC. GFP-LUC-labeled BRx68 CTCs (BRx68_luc) were FACS sorted into three morphometric subgroups as described above. Tumors were established by inoculation of 200 sorted cells in 100 μL of Matrigel containing PBS into the 4th MFP of 6–8-week-old female NOD/SCID-Gamma (NSG) mice supplemented with subcutaneous slow-release estrogen pills. Tumor growth was monitored every 2 weeks by in vivo Bioluminescence imaging using IVIS Lumina III (PerkinElmer, Waltham, MA, USA) following intraperitoneal injection of 100 μL of d-luciferin substrate (Syd Labs, Hopkinton, MA, USA).

### 2.8. Statistical Analysis

For morphometric investigations, statistical two-sided analysis was performed using R (Version 4.1.1, Boston, MA, USA). Groups were compared using Student’s *t*-test to determine if there is a significant difference between the means of the two groups. For in vivo experiments, statistical analysis was performed using GraphPad Prism Pro7. Mean ± SD of total efflux of bioluminescence of 4 to 5 mice per group were compared using unpaired two-tailed Student’s *t*-test. For all statistical analyses, *p*-values below 0.05 were considered statistically significant.

## 3. Results

### 3.1. CTCs Have Distinct Morphologies

**Figure 1:**
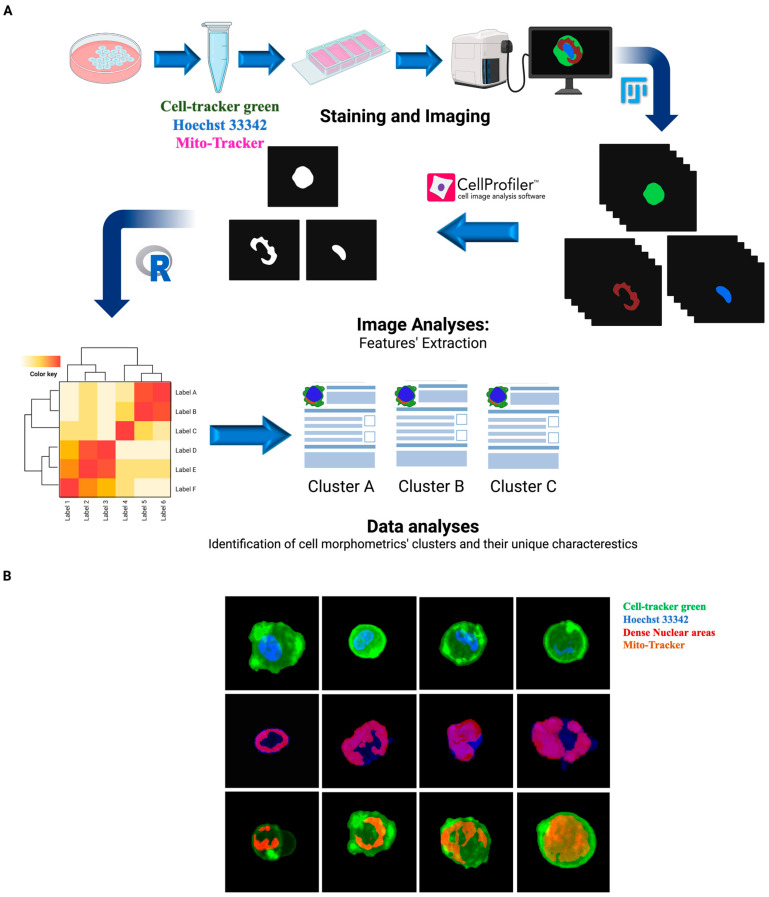
Morphometric analyses of CTCs. (**A**) Overview of the high-content image analysis workflow. (**B**) Digitally labelled images of single CTCs stained with CellTracker Green (Green), Hoechst 33342 (Blue), Mito-Tracker DeepRed (Magenta) and imaged using Z stack function of BZ-X800 (Keyence) at 40× magnification. Dense areas of the nucleus are labeled red.

Using previously established CTC lines from breast cancer patients [32], we identified different morphometric subgroups of CTCs using an automatic 3D imaging system, based on three-channel immunofluorescence (IF) staining followed by R-based high-content image data analyses (Figure 1A). IF corresponds to the nuclear dye (Hoechst 33342), the cytoplasmic dye (CellTracker Green), and the mitochondrial dye (Mito-Tracker-DeepRed). Initial microscopic observation showed that CTCs are heterogeneous in the volumes and shapes of whole cells and organelles, nucleus, mitochondria to cell ratio, and dense nuclear materials to nucleus ratios (Figure 1B). Principal component analyses (PCA) of high-content image data analyses of 500 single CTCs showed that cells cluster as one major group with two branches on both sides. Branching of cells out of the main cluster was primarily based on the cell surface texture.

**Figure 2:**
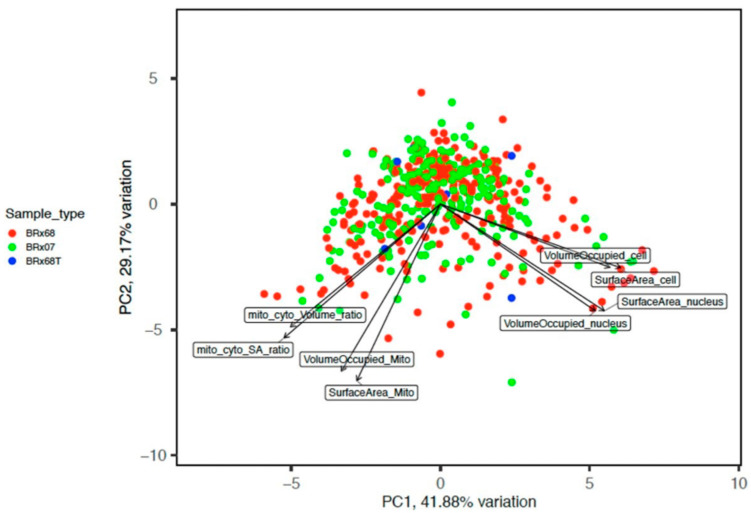
CTCs morphometric features. A PCA plot based on volumetric parameters and aspect ratios excluding texture and granularity (Main Features).

Whereas after removing texture and granularity out of the dimensionality reduction analyses, one of the branches was mainly segregated based on mitochondrial morphometric features, and another one was based on cell and nucleus features (Figure 2). 

**Figure 3:**
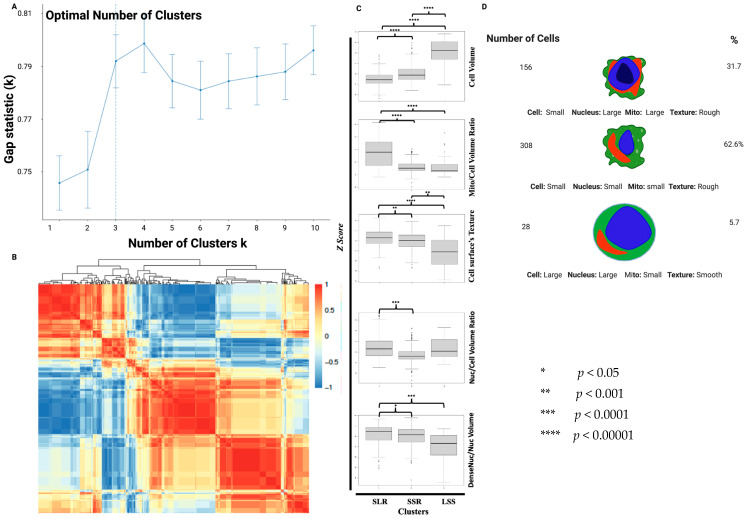
Morphometric characteristics of CTC subgroups. (**A**) Graph showing optimal number of clusters detected using the “fviz_nbclus” tool based on Main Features. (**B**) Heatmap showing clustering samples based on their morphometric profiles using only Main Features. (**C**) Boxplots represent Z scores of morphometric features across different CTC morphometric subgroups. (**D**) Cartoon depicting unique morphometric characteristics of different CTC subgroups with their proportions in the total population of cells.

Optimal clustering analyses using Main Features (all features except granularity and texture) showed that CTCs have three distinct groups (Figure 3A,B). We found that one group contains cells with small cell volume, large nucleus to cell (Nuc/Cell), dense nuclear areas to nucleus (DenseNuc/Nuc), and mitochondria to cell (Mito/Cell) volume ratios and rough texture of cell membrane. This group represents 32% of cells and is termed “Small Cell, Large Mitochondria, Rough Membrane” (SLR). Another group was rich in small cells with small Nuc/Cell, and Mito/Cell volume ratios and having a large DenseNuc/Nuc ratio and a rough texture of cell membrane. This group represents 62.6% of cells and is termed “Small Cell, Small Mitochondria, Rough Cell Membrane” (SSR), whereas the third group was rich in cells that are large in volume, with large Nuc/Cell and small Mito/Cell volume ratios and smooth texture of cell membrane. This group represents 5.7% of cells and is termed “Large Cell, Small Mitochondria, Smooth Cell Membrane” (LSS) (Figure 3C,D). 

The cell volumes of the SLR and SSR groups were significantly smaller than that of the LSS cells, and both SLR and SSR have rougher cell surface texture compared to LSS, which is even more obvious in SLR than that of the SSR (Figure 3C). Although both the SLR and SSR were relatively small, the Mito/Cell and the Nuc/Cell volume ratios of the SLR were significantly higher than those of the SSR. There was no significant difference in Nuc/Cell volume ratio between SLR and LSS. In addition to having relatively large nuclei, the SLR group seems to have the densest nuclei, followed by the SSR group and the LSS group (Figure 3C,D). 

### 3.2. SLR CTCs Divide Slowly In Vitro but Have Higher Tumorigenic Capabilities In Vivo

To test whether the three morphometrically distinct groups have different functions, we will need a larger number of cells to represent each group. Therefore, we optimized and validated a FACS strategy to sort cells that represent the three microscopically defined morphometric groups. We mainly focused on the cell size and Mito/Cell ratio as other features are hard to quantify using FACS. The first gating step based on CellTracker Green sorted the largest (large) and the smallest (small) 10% of the whole cell population. The second gating step based on Mito-Tracker DeepRed sorted the “small” fraction into low (lowest 30 to 40%) and high (highest 10%) mitochondria, resulting in a total of three fractions: “Small/Low”, “Small/High” and “Large” (Supplementary Figure S1A). 

**Figure 4:**
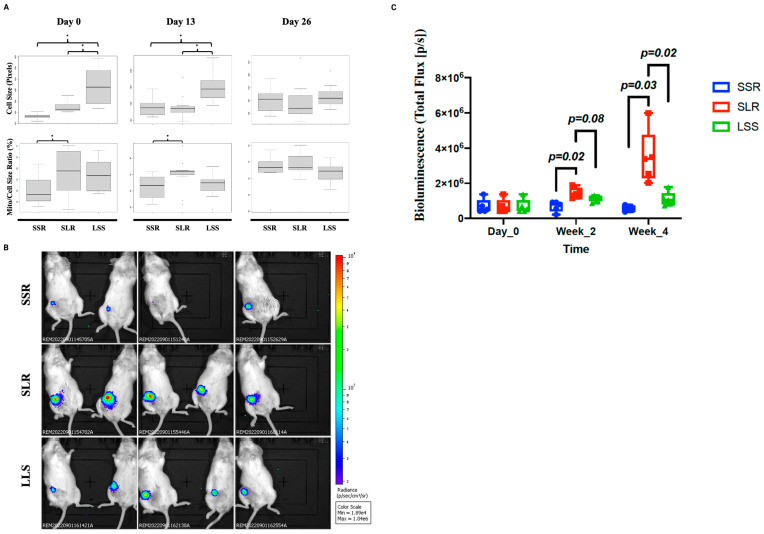
CTC morphometric subgroups preserve their morphologies in vitro and have distinct tumorgenicity in vivo. (**A**) Microscopically measured cell size and Mito/Cell size ratio of FACS sorted cells at day 0 of sorting and at days 13 and 26 days of ex vivo culture. Cell size was extracted from CellTracker Green 2D images using CellProfiler and presented in pixels as described in the methods. Mito/Cell size ratio (%) was calculated by dividing mitochondria size (pixels), as extracted from Mito-Tracker DeepRed 2D images, by cell size and then multiplying by 100. (**B**) In vivo imaging of NSG mice which are transplanted with BRx68_luc CTC subgroups into the 4th mammary fat pads at week 4 of transplantation. (**C**) Quantification of bioluminescence signals at different time points. Each dot represents the total Flux (p/s) from one mouse at a given time point. Mice were imaged at day 0, and weeks 2 and 4 post implantation. * *p* < 0.05.

Microscopic investigations of cells from these fractions confirmed that the “Small/High” and the “Small/Low” cells were smaller in size than that of the “Large” group. Moreover, the “Small/High” group and the “Large” group showed significantly higher Mito/Cell size ratio than that of the “Small/Low” group. These analyses showed that the FACS sorted fractions (“Small/High”, “Small/Low” and “Large”) mimic the SLR, SSR and LSS, respectively (Figure 4A). Therefore, these terms were used in this manuscript to refer to either microscopically defined groups or their respective FACS-sorted cell groups. 

We hypothesized that the SLR cells may have a higher metastatic ability. The small size may allow them to pass through narrow blood capillaries [41,42,43] and the high mitochondrial content may support energy-dependent metastatic steps [44] (Supplementary Figure S1B). Although FACS sorting provided enough cells for in vitro proliferation assay, it was not possible to get enough cells to perform a proper in vivo metastatic assay. Therefore, to test our hypothesis, we monitored morphometric features during CTC proliferation in vitro and tested their tumorigenicity in vivo. 

When cultured in vitro, the SLR cells showed a reduction in cell number from 1000 to 500 cells during the first two weeks, whereas the SSR cells showed the highest proliferation rate followed by the LSS cells (Supplementary Figure S1C). Microscopic imaging on day 13 showed that the morphometric characteristics of the three groups were preserved. Both SLR and SSR cells showed small cell sizes compared with the LSS group. Moreover, the Mito/Cell size ratio was significantly higher in the SLR than that of the SSR cells (Figure 4A). By day 26, the SLR cell proliferation rate increased from day 13 to what is comparable to other groups (Supplementary Figure S1C). The boost in SLR cell proliferation seems to have been caused by the emergence of other cell morphologies. Microscopic imaging at day 26 showed that SLR cells are no longer smaller than that of LSS cells and no longer have larger mitochondrial content compared with the SSR cells (Figure 4A). 

To test the tumorigenic capability potentially associated with cells with morphometric differences, we used BRx68 cells which are transduced with lentivirus-carrying luciferase (BRx68_luc) to allow in vivo monitoring of tumor growth. A FACS protocol was optimized to sort BRx68_luc cells based on their size and mitochondrial content (Supplementary Figure S2) and cells from 3 groups were sorted and 200 cells per mouse were injected into the mammary fat pads (MFP) of the female NSG mice. Interestingly, the SLR group showed the highest tumorigenic activity compared to the LSS and the SSR groups (Figure 4B). At week 2 post implantation, the SLR tumors showed significantly higher bioluminescence than that of the SSR group. There was no significant difference in the bioluminescence between SLR and LSS. By week 4, SLR tumors were significantly larger than that of the two other groups (Figure 4C). This data showed that despite a slower proliferation in vitro, the SLR cells have a higher tumorigenic capacity in vivo compared to the two other types. 

### 3.3. Single Cell RNA Sequencing Suggests That SLR Group May Have Stem Cell Properties

To understand molecular portraits of different CTC morphometric groups, single cells from each group were processed for RNA-seq analyses. Only cells with more than 5 million reads which are uniquely assigned to exonic regions of the human genome were included in the analyses. Small cells (SLR and SSR) were shown to be transcriptionally distinct from large cells (Supplementary Figure S3A). We next detected upregulated genes in the small groups (SLR and SSR combined) compared with the LSS group and used them for functional and signaling pathway enrichment analyses using nRichr. Small cells were shown to be metabolically active, where metabolism-related signaling pathways and cell processes were among the top enriched pathways/cell processes in the small group. Both Reactome 2016 and the Bioplanet 2019 databases showed that unfolded protein response signaling pathways are highly enriched in the small group, whereas cell senescence, the amino sugar and nucleotide sugar metabolism, lysosomes, thyroid and growth hormones, and phosphatidylinositol were top enriched pathways in the KEGG 2021 human database (Supplementary Figures S3B and S4, and Supplementary Tables S1–S3). In terms of ontologies, the GO Biological Process 2021 database showed that the Mitochondrial RNA metabolic process was the top enriched cell process (Supplementary Figure S3B and Table S4). Moreover, cell type analyses using PanglaoDB Augmented 2021 database suggest olfactory or urothelial cells with genes enriched in the small group (Supplementary Figure S3B and Table S5). 

**Figure 5:**
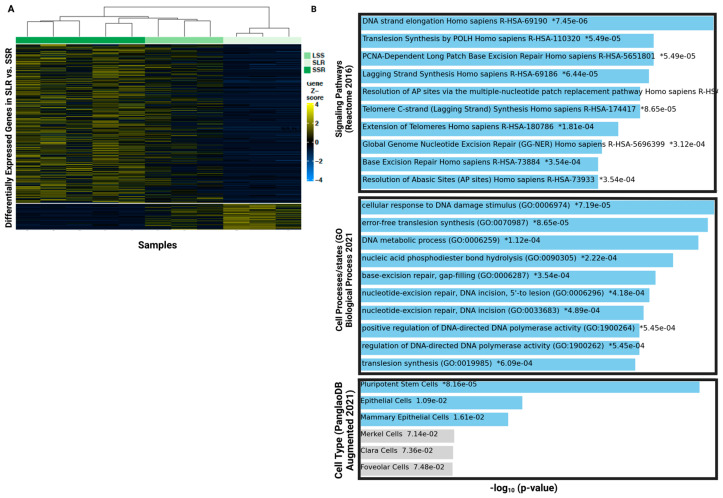
Single-cell RNA sequencing of BRx68 morphometric subgroups. (**A**) Heatmap showing clustering analyses of a total of 11 cells from BRx68 clusters based on supervised DEGs in the SLR vs. SSR cells (*n* = 636). (**B**) Bar graphs showing the top 10 enriched terms in Small vs. Large cells (n = 781 genes) and SLR vs. SSR cells (*n* = 79 genes) from the Reactome 2016 (signaling pathways), GO Biological Processes 2021 (cell processes/states) and the PanglaoDB Augmented 2021 (Cell types) databases. Enrichment analyses were performed using Enrichr based on upregulated genes. Colored bars correspond to terms with significant *p*-values (<0.05). An asterisk (*) next to a *p*-value indicates the term also has a significant adjusted *p*-value (<0.05).

Principal component analyses (PCA) showed no clear separation between SSR and SLR (Supplementary Figure S3A). However, clustering analyses supervised by differentially expressed genes (DEGs) in the SLR group compared with the SSR group showed that cells are clustered into their morphometric categories (Figure 5A). Enrichment analyses using Enrichr showed that the SLR group is enriched in pathways and cellular processes related to DNA replication, DNA repair and metabolism. The Reactome 2016, the Bioplanet 2019 and the KEGG 2021 human databases showed that DNA strand elongation/replication is the top enriched signaling pathway, followed by nucleotide excision and mismatch repair and glycosylation and N Glycan biosynthesis (Figure 5B and Figure S4 and Supplementary Tables S6–S8). Gene ontologies analyses using the GO biological processes database showed that cellular response to DNA damage stimulus, and DNA repair specific processes such as base-excision repair, gap-filling, nucleotide-excision repair, DNA incision, 5′-to lesion, post-replication repair, etc., are highly enriched in the SLR group (Figure 5B and Supplementary Table S9). Interestingly, cell type analyses suggest that DEGs of SLR cells are related to pluripotent stem cells, followed by mammary epithelial cells (Figure 5B and Supplementary Table S10). 

### 3.4. Expression of SLR Upregulated Genes in Primary Tumors Could Predict Clinical Prognosis

**Figure 6:**
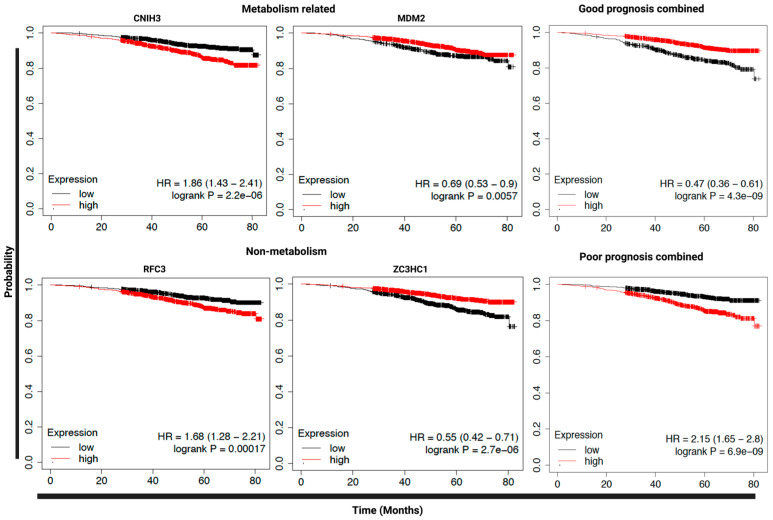
Prognostic values of upregulated genes in the SLR vs. SSR. Kaplan–Meier curves showing overall survival (OS) of ER^+^ breast cancer patients from the KM plotter database (2575 patients), based on their primary tumor expression. Kaplan–Meier curves show only genes with strongest association with OS (highest and lowest Hazard Ratio (HR)) in the metabolism-related group (top), non-metabolism group (bottom), and all genes associated with either good (*n* = 15 genes) (top) or poor (*n* = 19 genes) prognosis combined (bottom). *p*-values were determined via a log-rank test.

We next performed a series of survival analyses to explore whether increased primary tumor expression of SLR upregulated genes is predictive of poor survival in patients with ER^+^ breast cancer using RNA-seq data from the KM Plotter database (2575 patients). This was done to determine whether SLR-upregulated genes are higher in more biologically aggressive tumors and whether they have value as predictive biomarkers for disease progression in patients. We found that 34 of 79 SLR upregulated genes were significantly associated with overall survival (OS) of ER^+^ patients (*p* < 0.05, hazard ratio (HR) 0.5 to 1.9; Supplementary Table S11). Combining all 19 genes associated with poor prognosis and the 15 genes associated with good prognosis increased the power for predicting patient prognosis in this cohort (poor prognosis combined: HR = 2.3, *p* < 0.00001, good prognosis combined: HR = 0.47, *p* < 0.00001) (Figure 6).

Interestingly, 6 out of the 34 genes are known to be involved in metabolism, whereas other genes are playing roles in DNA replication, DNA repair, cell cycle, proliferation, migration, and gene transcription and translation, termed collectively in this manuscript as “non-metabolism”. Five genes out of the 6 metabolism-related genes are significantly associated with poor prognosis, with CNIH3 showing the highest hazard ratio (HR= 1.9, *p* < 0.00001) and only one gene, MDM2 (HR = 0.7, *p* = 0.005), associated with good prognosis (Figure 6 and Supplementary Table S11). Whereas 15 of the non-metabolism genes are significantly associated with good prognosis with the lowest HR of 0.5 for the ZC3HC1gene (*p* < 0.00001) and only 6 genes are associated with poor prognosis with RFC3 which has the highest HR of 1.7 (*p* = 0.0001) (Figure 6 and Supplementary Table S11). This suggests that most of the non-metabolism genes in our list are negatively regulating cell proliferation and migration, which potentially explain the slow proliferation rate of the SLR cells in vitro. 

## 4. Discussion

CTCs attracted widespread interest as a minimally invasive tumor marker used for cancer diagnosis, prognosis, and prediction of therapeutic response in breast cancer and other tumors. Moreover, it can provide novel insights into metastasis and potential therapeutic targets [3,45,46,47]. Recent studies have shown that CTCs present a clonal heterogeneity with only a subset of the cells having the ability to initiate metastasis [32,48]. However, an in-depth look at the cellular morphology that is associated with this heterogeneity has yet to be done. 

In this study, we focused on the morphometric heterogeneity of two patient-derived CTC lines and found that the total CTC population contains three different morphological phenotypes with distinct transcriptomic profiles and tumorigenic capacities. Our results align with previous studies showing links between specific morphological structures in primary tumors and gene expression profiles and signaling pathways [49]. Our study also found that cell morphological traits were preserved for up to 4 weeks in culture, in concordance with a previous report that showed similar traits in single-cell clones [50]. This is the first study to detect CTC morphometric phenotypes with associated transcriptomic profiles and tumorigenic capabilities, suggesting the potential use of cellular morphometric properties to indicate cellular function. 

We found that cell surface texture was the key driver in determining morphological subsets, followed by cell volume/size. Two small CTC groups have rough surfaces whereas the large group has a smooth surface. Previous studies have shown that high-metastatic cancer cells exhibit rough surfaces to increase cell migration and invasion [51]. For example, some CTCs have been shown to have a distinct form of membrane structure called Microtentacles (McTNs) that are strongly correlated with metastatic ability [52]. However, we were not able to perform a classical in vivo metastatic assay via intracardiac or intravenous injections because of the limited number of cells. In addition, FACS-sorted groups only represented Mito/Cyto ratio and cell size; thus, further experiments can be done to look at Nuc/Cell and DensNuc and their relationship with metastatic potential. 

Cell size has long been considered a key predictor of cancer cell behavior, however, with conflicting views. Smaller cells from osteosarcoma and colorectal cancer cell lines have been shown to have enhanced metastatic capacity [43,53]. On the contrary, larger tumor cells have also been shown to have enhanced metastatic potentials due to their ability to easily get arrested in the blood capillaries of certain organs [54]. Our study found that cell size is not the only determining factor for tumorigenicity, as the SSR group had the weakest tumorgenicity in vivo whereas SLR has the highest. 

A unique feature of the SLR cell group is that they have a larger Nuc/Cell ratio, which was shown to be linked to cell aggressiveness. This aligns with previous studies showing large nuclear diameter as a key morphologic characteristic associated with shorter patient survival [55] and aggressiveness [50,56]. In addition, the nuclei of the SLR cells were relatively denser (high DenseNuc/Nuc ratio) than that of the other two cell phenotypes. It was previously shown that dense foci in the nucleus stained by Hoechst 33342 are representative of heterochromatin [57], which is associated with a poor patient prognosis. This study did not examine this feature in FACS-sorted groups, making further studies necessary, such as single-cell Hi-C analysis of CTCs.

The high Mito/Cell ratio in the SLR cells indicates metabolic reprogramming, a hallmark of cancer. This provides cells with energy and precursor molecules needed for cell proliferation and metastasis [58]. It has been shown that CTCs with elevated glucose metabolism are more abundant in metastatic patients and have a stronger association with metastasis than CTCs EMT subtypes [59]. Cells with high mitochondrial content have been shown to have enhanced tumorigenesis and resistance to paclitaxel, resulting in little or no DNA damage [44]. This agrees with our scRNA-seq data which showed that genes in DNA repair machinery, and DNA damage responses are highly expressed in SLR cells, which suggests that SLR cells may exhibit resistance to therapy but needs further validation. 

scRNA-seq-based cell type analyses recommend that DEGs in SLR cells are shared with that from pluripotent stem cells, suggesting the possibility of sharing properties with cancer stem cells (CSCs). CSCs are known to be tumorigenic, can self-renew and differentiate into non-CSC cells. They have been shown to have increased DNA damage responses and repair mechanisms [60,61], high mitochondrial content [44], higher levels of McTN [62] and often slow cycling. Despite high tumorigenicity in vivo, SLR cells had a slow cell proliferation in vitro with preservation of their unique morphometric characteristics within 4 weeks. Intriguingly, after 4 weeks in culture, SLR cells lost these features, suggesting the possibility of differentiating into other phenotypes over time. Further studies, such as stem cell marker analysis and self-renewal evaluation, are needed to confirm this hypothesis. The presence of CTCs bearing CSC properties was previously reported and was shown to be tumorigenic and more closely associated with prognosis and therapy resistance than total CTCs [63,64]. Indeed, our survival analyses found that nearly half of the upregulated genes in the SLR cells are associated with ER^+^ breast cancer patients’ survival, indicating potential clinical relevance. 

Our new findings provide a simple image-based identification of CTC subpopulations with elevated aggressiveness. Considering the potential future advances in artificial intelligence-based imaging analysis, our finding is expected to provide a more accurate prediction of patient survival and therapy response than total CTC numbers in breast cancer, and likely in other epithelial cancers as well. Despite a clear association of SLR cells with higher tumorigenic potential in vivo, most of our morphometric characterization is done with CTCs cultured ex vivo. It will be interesting with follow-up studies using freshly isolated CTCs to confirm the clinical utility. Although we have not specifically investigated morphometric heterogeneity of the primary tumor or metastatic microenvironments, it was reported that breast cancer metastasis could modulate the morphometric architecture of the metastasis microenvironment such as osteoclasts in bone metastases [65]. The detection of SLR cells’ morphometric and transcriptomic profiles opens opportunities for targeted cancer treatment. Potential targets within mitochondria, such as BCL-2 family inhibitors and HSP90 that are only expressed in cancer cells [66,67,68], or specific DNA repair components, could improve therapy outcomes. The enrichment of highly tumorigenic cells in SLR cells also has potential in precision oncology to target specific pathways or use them for drug resistance screening. This study is the first to provide an image-based identification of aggressive CTC subtypes and holds promise for more accurate cancer management in the future.

## 5. Conclusions

Using previously established CTC lines from breast cancer patients, we developed an imaging-based pipeline to identify morphometric distinct subgroups of CTCs. Combined with in vivo tumorigenic assay and RNA-seq analysis, we found that a subgroup of CTCs with small cell sizes, large mitochondria and rough membrane texture have enhanced tumorigenesis and higher expression of genes related to stemness and poor survival correlation, which is expected to provide a more accurate prediction of patient survival and therapy response than total CTC numbers.

## Data Availability

Data are available upon request.

## Supplementary Materials

The following supporting information can be downloaded at: https://www.mdpi.com/article/10.3390/cancers15102669/s1, Figure S1: in vitro functional characterization of CTCs’ morphometric clusters. Figure S2: FACS protocol for sorting BRx68-luc CTCs’ morphometric clusters. Figure S3: Single-cell RNA sequencing of BRx68 morphometric clusters. Figure S4: Signaling pathways enrichment analyses of CTCs phenotypes. Table S1: Signaling_Reactome_2016_Small_vs_Large_RightClusterOut_UpLog2_P0.05. Table S2: Signaling_Bioplanet_2019_Samll_VS_Large_RightClusterOut_UpLog2_P0.05. Table S3: Signaling_KEGG_Small_Vs_Large_RightClusterOut_UpLog2_P0.05. Table S4: Ontologies_GO_Biological_Process_2021_small_vs_large_RightClusterOut_UpLog2_P0.05. Table S5: Celltype_PanGlaoDB_Small_Vs_Large_RightClusrterOut_UpLog2_P0.05. Table S6: Signaling_Reactome2016_SLR_Vs_SSR_RightClusterOut_LSSOut_UpLog2_P0.05. Table S7: Signaling_BioPlanet_SLR_Vs_SSR_RightClusterOut_LSSOut_UpLog2_P0.05. Table S8: Signaling_KEGG_human2021_SLR_vs_SSR_RightClusterOut_LSS)out_upLog2P0.05. Table S9: _Ontologies_GO_SLR_Vs_SSR_RightClusterOut_LSSOut_UpLog2_P0.05. Table S10: CellType_PanglaoDB_Augmented_2021_table_SLR_Vs_SSR_RightClusterOut_UpLog2_P0.05. Table S11: SLR_Vs_SSR_UpLog2_P0.05_Survival_analyses2.

## Abbreviations

CTC	circulating tumor cell
SLR	small cell, large mitochondria, rough membrane
SSR	small cell, small mitochondria, rough membrane
LSS	large cell, small mitochondria, smooth membrane
KM Plotter	Kaplan–Meier plotter
PFS	progression-free survival
OS	overall survival
FACS	fluorescence-activated cell sorting
IF	immunofluorescence
PBS	phosphate-buffered saline
BSA	bovine serum albumin
IQR	interquartile range
FSC	forward scatter (area/height)
SSC	side scatter (area/height)
FDR	false discovery rate
MFP	mammary fat pad
NSG mice	NOD/SCID-Gamma
HR	hazardous ratio
EMT	epithelial–mesenchymal transition
CSC	cancer stem cell

## References

[1] Siegel R.L., Miller K.D., Fuchs H.E., Jemal A. (2021). Cancer Statistics, 2021. CA Cancer J. Clin..

[2] Nguyen D.X., Bos P.D., Massagué J. (2009). Metastasis: From dissemination to organ-specific colonization. Nat. Rev. Cancer.

[3] Cristofanilli M., Budd G.T., Ellis M.J., Stopeck A., Matera J., Miller M.C., Reuben J.M., Doyle G.V., Allard W.J., Terstappen L.W. (2004). Circulating tumor cells, disease progression, and survival in metastatic breast cancer. N. Engl. J. Med..

[4] Andreopoulou E., Cristofanilli M. (2010). Circulating tumor cells as prognostic marker in metastatic breast cancer. Expert Rev. Anticancer.

[5] Cristofanilli M., Hayes D.F., Budd G.T., Ellis M.J., Stopeck A., Reuben J.M., Doyle G.V., Matera J., Allard W.J., Miller M.C. (2005). Circulating tumor cells: A novel prognostic factor for newly diagnosed metastatic breast cancer. J. Clin. Oncol..

[6] Horton C.E., Kamal M., Leslie M., Zhang R., Tanaka T., Razaq M. (2018). Circulating Tumor Cells Accurately Predicting Progressive Disease After Treatment in a Patient with Non-small Cell Lung Cancer Showing Response on Scans. Anticancer Res..

[7] De Bono J.S., Scher H.I., Montgomery R.B., Parker C., Miller M.C., Tissing H., Doyle G.V., Terstappen L.W., Pienta K.J., Raghavan D. (2008). Circulating tumor cells predict survival benefit from treatment in metastatic castration-resistant prostate cancer. Clin. Cancer Res..

[8] Cohen S.J., Punt C.J., Iannotti N., Saidman B.H., Sabbath K.D., Gabrail N.Y., Picus J., Morse M., Mitchell E., Miller M.C. (2008). Relationship of circulating tumor cells to tumor response, progression-free survival, and overall survival in patients with metastatic colorectal cancer. J. Clin. Oncol..

[9] Cohen S.J., Punt C.J., Iannotti N., Saidman B.H., Sabbath K.D., Gabrail N.Y., Picus J., Morse M.A., Mitchell E., Miller M.C. (2009). Prognostic significance of circulating tumor cells in patients with metastatic colorectal cancer. Ann. Oncol..

[10] Bidard F.C., Peeters D.J., Fehm T., Nolé F., Gisbert-Criado R., Mavroudis D., Grisanti S., Generali D., Garcia-Saenz J.A., Stebbing J. (2014). Clinical validity of circulating tumour cells in patients with metastatic breast cancer: A pooled analysis of individual patient data. Lancet Oncol..

[11] Zhang L., Riethdorf S., Wu G., Wang T., Yang K., Peng G., Liu J., Pantel K. (2012). Meta-analysis of the prognostic value of circulating tumor cells in breast cancer. Clin. Cancer Res..

[12] Giordano A., Egleston B.L., Hajage D., Bland J., Hortobagyi G.N., Reuben J.M., Pierga J.Y., Cristofanilli M., Bidard F.C. (2013). Establishment and validation of circulating tumor cell-based prognostic nomograms in first-line metastatic breast cancer patients. Clin. Cancer Res..

[13] Pierga J.Y., Hajage D., Bachelot T., Delaloge S., Brain E., Campone M., Dieras V., Rolland E., Mignot L., Mathiot C. (2012). High independent prognostic and predictive value of circulating tumor cells compared with serum tumor markers in a large prospective trial in first-line chemotherapy for metastatic breast cancer patients. Ann. Oncol..

[14] Smerage J.B., Barlow W.E., Hortobagyi G.N., Winer E.P., Leyland-Jones B., Srkalovic G., Tejwani S., Schott A.F., O’Rourke M.A., Lew D.L. (2014). Circulating tumor cells and response to chemotherapy in metastatic breast cancer: SWOG S0500. J. Clin. Oncol..

[15] Wallwiener M., Hartkopf A.D., Baccelli I., Riethdorf S., Schott S., Pantel K., Marmé F., Sohn C., Trumpp A., Rack B. (2013). The prognostic impact of circulating tumor cells in subtypes of metastatic breast cancer. Breast Cancer Res. Treat..

[16] Wallwiener M., Riethdorf S., Hartkopf A.D., Modugno C., Nees J., Madhavan D., Sprick M.R., Schott S., Domschke C., Baccelli I. (2014). Serial enumeration of circulating tumor cells predicts treatment response and prognosis in metastatic breast cancer: A prospective study in 393 patients. BMC Cancer.

[17] Liu M.C., Shields P.G., Warren R.D., Cohen P., Wilkinson M., Ottaviano Y.L., Rao S.B., Eng-Wong J., Seillier-Moiseiwitsch F., Noone A.M. (2009). Circulating tumor cells: A useful predictor of treatment efficacy in metastatic breast cancer. J. Clin. Oncol..

[18] Hayes D.F., Cristofanilli M., Budd G.T., Ellis M.J., Stopeck A., Miller M.C., Matera J., Allard W.J., Doyle G.V., Terstappen L.W. (2006). Circulating tumor cells at each follow-up time point during therapy of metastatic breast cancer patients predict progression-free and overall survival. Clin. Cancer Res..

[19] Riethdorf S., Soave A., Rink M. (2017). The current status and clinical value of circulating tumor cells and circulating cell-free tumor DNA in bladder cancer. Transl. Androl. Urol..

[20] Yu M., Bardia A., Wittner B.S., Stott S.L., Smas M.E., Ting D.T., Isakoff S.J., Ciciliano J.C., Wells M.N., Shah A.M. (2013). Circulating breast tumor cells exhibit dynamic changes in epithelial and mesenchymal composition. Science.

[21] Banys M., Krawczyk N., Becker S., Jakubowska J., Staebler A., Wallwiener D., Fehm T., Rothmund R. (2012). The influence of removal of primary tumor on incidence and phenotype of circulating tumor cells in primary breast cancer. Breast Cancer Res. Treat..

[22] Fehm T., Hoffmann O., Aktas B., Becker S., Solomayer E.F., Wallwiener D., Kimmig R., Kasimir-Bauer S. (2009). Detection and characterization of circulating tumor cells in blood of primary breast cancer patients by RT-PCR and comparison to status of bone marrow disseminated cells. Breast Cancer Res..

[23] Fehm T., Müller V., Aktas B., Janni W., Schneeweiss A., Stickeler E., Lattrich C., Löhberg C.R., Solomayer E., Rack B. (2010). HER2 status of circulating tumor cells in patients with metastatic breast cancer: A prospective, multicenter trial. Breast Cancer Res. Treat..

[24] Jordan N.V., Bardia A., Wittner B.S., Benes C., Ligorio M., Zheng Y., Yu M., Sundaresan T.K., Licausi J.A., Desai R. (2016). HER2 expression identifies dynamic functional states within circulating breast cancer cells. Nature.

[25] Aceto N., Toner M., Maheswaran S., Haber D.A. (2015). En Route to Metastasis: Circulating Tumor Cell Clusters and Epithelial-to-Mesenchymal Transition. Trends Cancer.

[26] Miyamoto D.T., Zheng Y., Wittner B.S., Lee R.J., Zhu H., Broderick K.T., Desai R., Fox D.B., Brannigan B.W., Trautwein J. (2015). RNA-Seq of single prostate CTCs implicates noncanonical Wnt signaling in antiandrogen resistance. Science.

[27] Babayan A., Hannemann J., Spoetter J., Mueller V., Pantel K., Joosse S.A. (2013). Heterogeneity of estrogen receptor expression in circulating tumor cells from metastatic breast cancer patients. PLoS ONE.

[28] Papadaki M.A., Kallergi G., Zafeiriou Z., Manouras L., Theodoropoulos P.A., Mavroudis D., Georgoulias V., Agelaki S. (2014). Co-expression of putative stemness and epithelial-to-mesenchymal transition markers on single circulating tumour cells from patients with early and metastatic breast cancer. BMC Cancer.

[29] Lee C.H., Hsieh J.C., Wu T.M., Yeh T.S., Wang H.M., Lin Y.C., Chen J.S., Lee C.L., Huang W.K., Hung T.M. (2019). Baseline circulating stem-like cells predict survival in patients with metastatic breast Cancer. BMC Cancer.

[30] Alizadeh E., Castle J., Quirk A., Taylor C.D., Xu W., Prasad A. (2020). Cellular morphological features are predictive markers of cancer cell state. Comput. Biol. Med..

[31] Ligthart S.T., Coumans F.A.W., Bidard F.-C., Simkens L.H.J., Punt C.J.A., De Groot M.R., Attard G., De Bono J.S., Pierga J.-Y., Terstappen L.W.M.M. (2013). Circulating Tumor Cells Count and Morphological Features in Breast, Colorectal and Prostate Cancer. PLoS ONE.

[32] Yu M., Bardia A., Aceto N., Bersani F., Madden M.W., Donaldson M.C., Desai R., Zhu H., Comaills V., Zheng Z. (2014). Cancer therapy. Ex vivo culture of circulating breast tumor cells for individualized testing of drug susceptibility. Science.

[33] Klotz R., Thomas A., Teng T., Han S.M., Iriondo O., Li L., Restrepo-Vassalli S., Wang A., Izadian N., MacKay M. (2020). Circulating Tumor Cells Exhibit Metastatic Tropism and Reveal Brain Metastasis Drivers. Cancer Discov..

[34] Zhang Y.T., Dong M., Xu P.P., Cai J.H., Liu S.H., Gao Y.B., Wang L.B., Li J., Jiang H., Wang J.D. (2022). Aptamer-mediated DNA concatemer functionalized magnetic nanoparticles for reversible capture and release of circulating tumor cells. Colloids Surf. B Biointerfaces.

[35] Stirling D.R., Swain-Bowden M.J., Lucas A.M., Carpenter A.E., Cimini B.A., Goodman A. (2021). CellProfiler 4: Improvements in speed, utility and usability. BMC Bioinform..

[36] Blighe K., Lun A. (2022). PCAtools: Everything Principal Components Analysis. https://github.com/kevinblighe/PCAtools.

[37] Liao Y., Smyth G.K., Shi W. (2014). featureCounts: An efficient general purpose program for assigning sequence reads to genomic features. Bioinformatics.

[38] Love M.I., Huber W., Anders S. (2014). Moderated estimation of fold change and dispersion for RNA-seq data with DESeq2. Genome Biol..

[39] Gu Z., Eils R., Schlesner M. (2016). Complex heatmaps reveal patterns and correlations in multidimensional genomic data. Bioinformatics.

[40] Kuleshov M.V., Jones M.R., Rouillard A.D., Fernandez N.F., Duan Q., Wang Z., Koplev S., Jenkins S.L., Jagodnik K.M., Lachmann A. (2016). Enrichr: A comprehensive gene set enrichment analysis web server 2016 update. Nucleic Acids Res..

[41] Lohse C.M., Cheville J.C., Blute M.L., Zincke H., Weaver A.L. (2002). Comparison of standardized and nonstandardized nuclear grade of renal cell carcinoma to predict outcome among 2,042 patients. Am. J. Clin. Pathol..

[42] Leleu X., Genevieve F., Guieze R., Duhamel A., Andrieux J., Berthon C., Godon A., Prat-Lesaffre S., Depil S., Lai J.L. (2005). Irregular nuclear shape of bone marrow plasma cells defines a multiple myeloma subgroup related to hypodiploidy and to short survival. Leuk. Res..

[43] Mu L., Huang K., Hu Y., Yan C., Li X., Tao D., Gong J., Qin J. (2017). Small-sized colorectal cancer cells harbor metastatic tumor-initiating cells. Oncotarget.

[44] Farnie G., Sotgia F., Lisanti M.P. (2015). High mitochondrial mass identifies a sub-population of stem-like cancer cells that are chemo-resistant. Oncotarget.

[45] Wu S., Zhao S., Cui D., Xie J. (2022). Advances in the Biology, Detection Techniques, and Clinical Applications of Circulating Tumor Cells. J. Oncol..

[46] Banys-Paluchowski M., Krawczyk N., Fehm T. (2016). Potential Role of Circulating Tumor Cell Detection and Monitoring in Breast Cancer: A Review of Current Evidence. Front. Oncol..

[47] Rack B., Schindlbeck C., Jückstock J., Andergassen U., Hepp P., Zwingers T., Friedl T.W.P., Lorenz R., Tesch H., Fasching P.A. (2014). Circulating tumor cells predict survival in early average-to-high risk breast cancer patients. J. Natl. Cancer Inst..

[48] Alix-Panabières C., Mader S., Pantel K. (2017). Epithelial-mesenchymal plasticity in circulating tumor cells. J. Mol. Med..

[49] Denisov E.V., Litviakov N.V., Zavyalova M.V., Perelmuter V.M., Vtorushin S.V., Tsyganov M.M., Gerashchenko T.S., Garbukov E.Y., Slonimskaya E.M., Cherdyntseva N.V. (2014). Intratumoral morphological heterogeneity of breast cancer: Neoadjuvant chemotherapy efficiency and multidrug resistance gene expression. Sci. Rep..

[50] Wu P.-H., Gilkes D.M., Phillip J.M., Narkar A., Cheng T.W.-T., Marchand J., Lee M.-H., Li R., Wirtz D. (2020). Single-cell morphology encodes metastatic potential. Sci. Adv..

[51] Suzuki N., Frapart M., Grdina D.J., Meistrich M.L., Withers H.R. (1977). Cell cycle dependency of metastatic lung colony formation. Cancer Res..

[52] Thompson K.N., Ju J.A., Ory E.C., Pratt S.J.P., Lee R.M., Mathias T.J., Chang K.T., Lee C.J., Goloubeva O.G., Bailey P.C. (2022). Microtubule disruption reduces metastasis more effectively than primary tumor growth. Breast Cancer Res..

[53] Muff R., Nigg N., Gruber P., Walters D., Born W., Fuchs B. (2007). Altered morphology, nuclear stability and adhesion of highly metastatic derivatives of osteoblast-like SAOS-2 osteosarcoma cells. Anticancer Res..

[54] Lu X., Kang Y. (2010). Organ-specific enhancement of metastasis by spontaneous ploidy duplication and cell size enlargement. Cell Res..

[55] Seili-Bekafigo I., Valkovic T., Babarović E., Duletić-Načinović A., Jonjić N. (2013). Myeloma cell morphology and morphometry in correlation with clinical stages and survival. Diagn. Cytopathol..

[56] Da Q., Deng S., Li J., Yi H., Huang X., Yang X., Yu T., Wang X., Liu J., Duan Q. (2022). Quantifying the cell morphology and predicting biological behavior of signet ring cell carcinoma using deep learning. Sci. Rep..

[57] Imai R., Nozaki T., Tani T., Kaizu K., Hibino K., Ide S., Tamura S., Takahashi K., Shribak M., Maeshima K. (2017). Density imaging of heterochromatin in live cells using orientation-independent-DIC microscopy. Mol. Biol. Cell.

[58] Hanahan D., Weinberg R.A. (2011). Hallmarks of cancer: The next generation. Cell.

[59] Chen J., Ye C., Dong J., Cao S., Hu Y., Situ B., Xi X., Qin S., Xu J., Cai Z. (2020). Metabolic classification of circulating tumor cells as a biomarker for metastasis and prognosis in breast cancer. J. Transl. Med..

[60] Desai A., Webb B., Gerson S.L. (2014). CD133+ cells contribute to radioresistance via altered regulation of DNA repair genes in human lung cancer cells. Radiother. Oncol..

[61] Wu J., Lai G., Wan F., Xiao Z., Zeng L., Wang X., Ye F., Lei T. (2012). Knockdown of checkpoint kinase 1 is associated with the increased radiosensitivity of glioblastoma stem-like cells. Tohoku J. Exp. Med..

[62] Charpentier M.S., Whipple R.A., Vitolo M.I., Boggs A.E., Slovic J., Thompson K.N., Bhandary L., Martin S.S. (2014). Curcumin targets breast cancer stem-like cells with microtentacles that persist in mammospheres and promote reattachment. Cancer Res..

[63] Aktas B., Tewes M., Fehm T., Hauch S., Kimmig R., Kasimir-Bauer S. (2009). Stem cell and epithelial-mesenchymal transition markers are frequently overexpressed in circulating tumor cells of metastatic breast cancer patients. Breast Cancer Res..

[64] Fan S.T., Yang Z.F., Ho D.W., Ng M.N., Yu W.C., Wong J. (2011). Prediction of posthepatectomy recurrence of hepatocellular carcinoma by circulating cancer stem cells: A prospective study. Ann. Surg..

[65] Badraoui R., Ben-Nasr H., Amamou S., El-May M.V., Rebai T. (2014). Walker 256/B malignant breast cancer cells disrupt osteoclast cytomorphometry and activity in rats: Modulation by α-tocopherol acetate. Pathol. Res. Pract..

[66] Kang B.H., Plescia J., Dohi T., Rosa J., Doxsey S.J., Altieri D.C. (2007). Regulation of tumor cell mitochondrial homeostasis by an organelle-specific Hsp90 chaperone network. Cell.

[67] Mootha V.K., Bunkenborg J., Olsen J.V., Hjerrild M., Wisniewski J.R., Stahl E., Bolouri M.S., Ray H.N., Sihag S., Kamal M. (2003). Integrated analysis of protein composition, tissue diversity, and gene regulation in mouse mitochondria. Cell.

[68] Kutuk O., Letai A. (2008). Alteration of the mitochondrial apoptotic pathway is key to acquired paclitaxel resistance and can be reversed by ABT-737. Cancer Res..

