# Supplementary material for: An Image-Based Identification of Aggressive Breast Cancer Circulating Tumor Cell Subtypes
Source: Cancers (Basel). 2023 May 9;15(10):2669. doi: 10.3390/cancers15102669 (PMC10216536; doi:10.3390/cancers15102669)
Supplement: Supplementary file 1 [file cancers-15-02669-s001.zip › cancers-2354917-supplementary.pdf]

Supplementary Figures

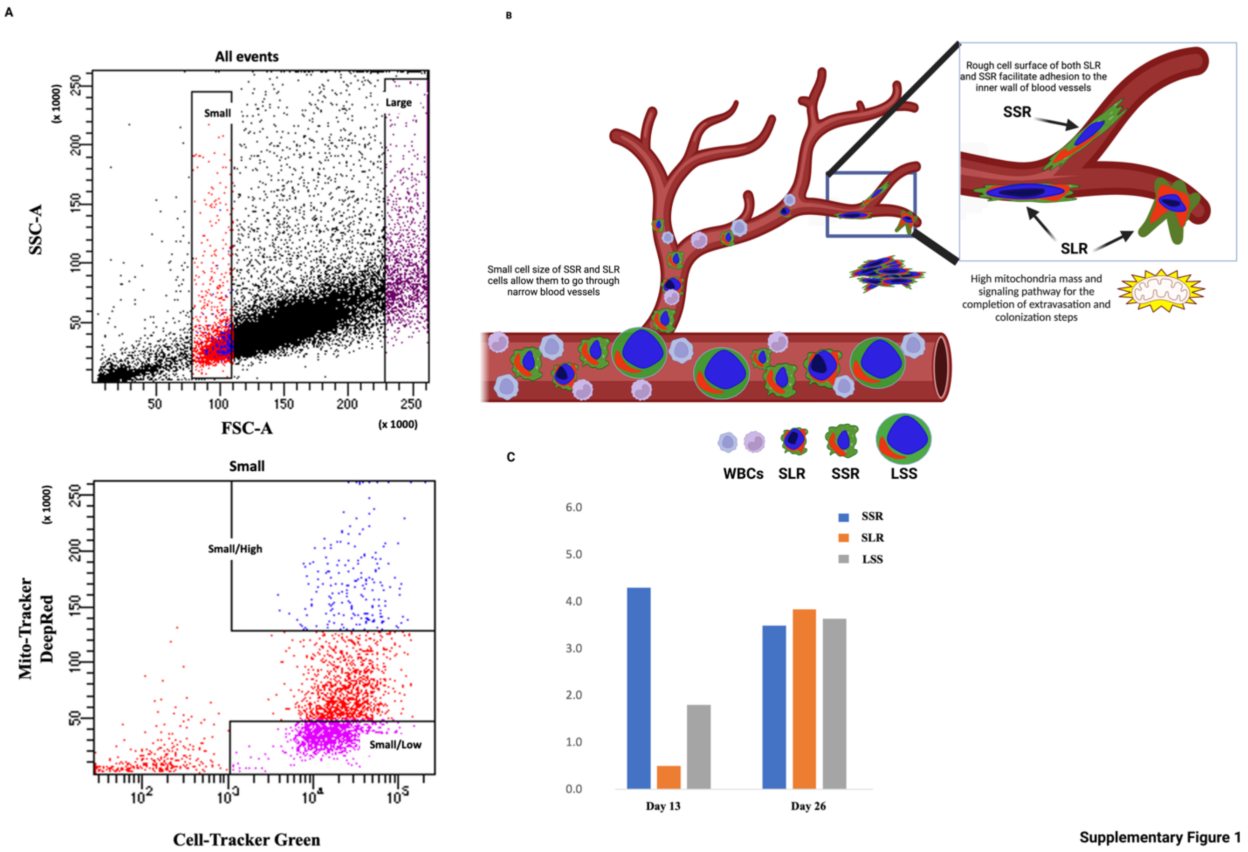

Suppl Figure S1: *in vitro* functional characterization of morphometric subgroups of CTCs. A) FACS gating allows for sorting BRx68 cells into their microscopically defined morphometric clusters. Cells were sorted following two gating steps: first (top) is to determine "Small" and "Large" groups based on the Forward Scatter-Area and Side Scatter-Area; second (bottom) is the "Small" group was sorted into "Small/High" and "Small/Low" based on the Cell-Tracker green and Mito-Tracker DeepRed. **B)** Cartoon depicting the hypothesis that the morphometric characteristics of SLR cells make them fit for the completion of the metastatic cascades. C) Bargraph showing cell proliferation rate of CTCs subgroups over 26 days. Graph shows the ratio of total cell numbers at one time point divided by that at the previous time point.

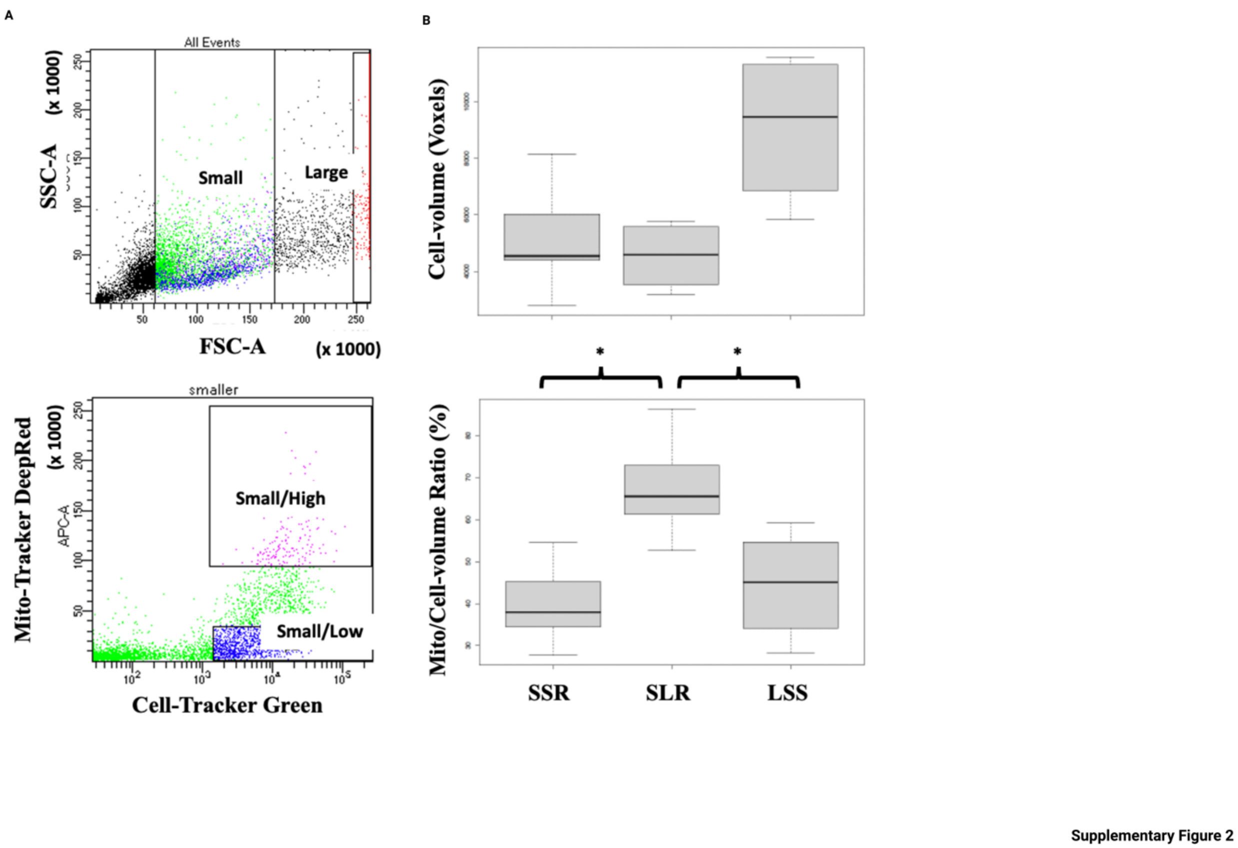

Suppl Figure S2: FACS protocol for sorting BRx68-luc CTC morphometric subgroups. A) FACS gating allows for sorting BRx68-luc cells into their microscopically defined morphometric subgroups as detailed in suppl. Fig 1. **B)** Microscopically measured cell volume and Mito/Cell volume ratio of FACS sorted cells. Cell volume was extracted in voxels from 3D images of Cell-Tracker green. Mito/Cell vol ratio was calculated by dividing mitochondria volume (voxels), as extracted from MitoTracker DeepRed 3D images, by cell volume then multiplying by 100.

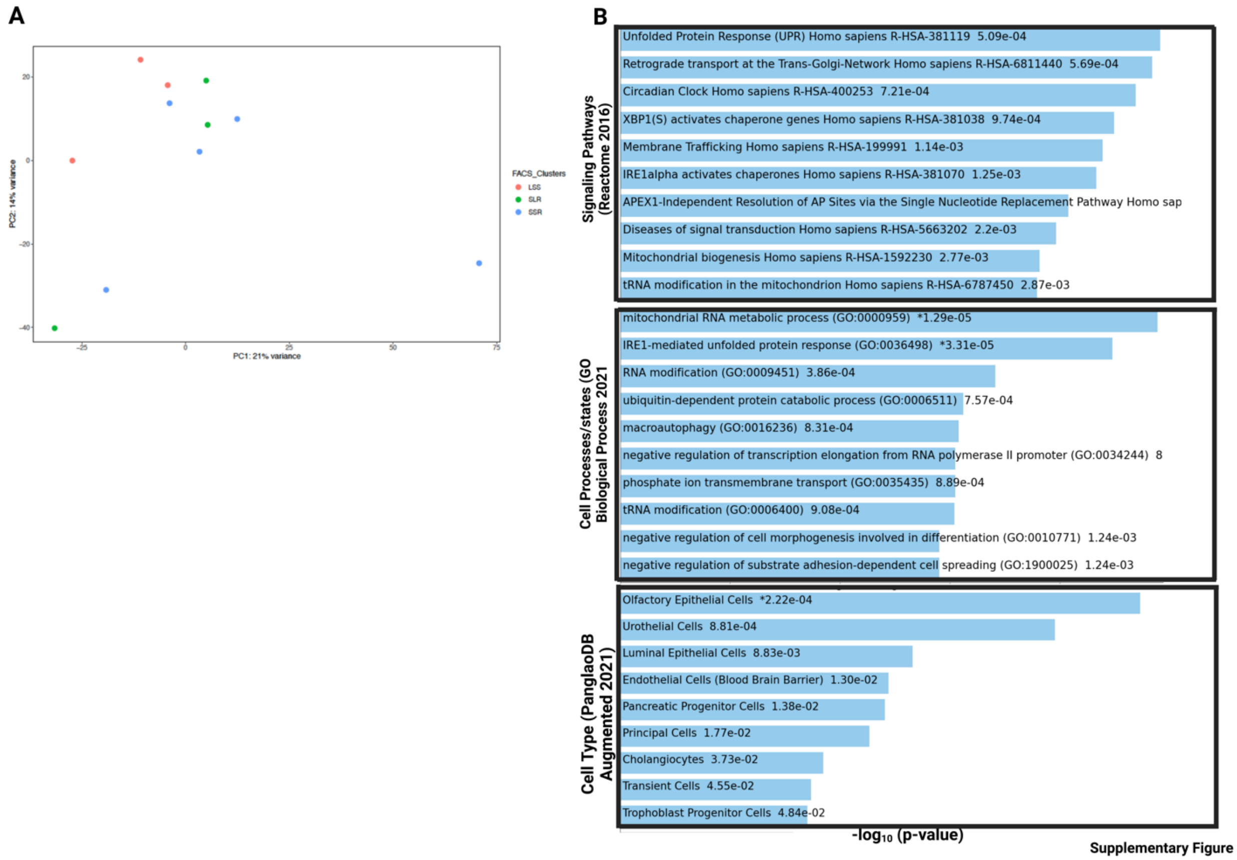

Supplementary Figure 3

Suppl Figure S3: Single cell RNA sequencing of BRx68 CTC morphometric subgroups. A) Principal component analysis (PCA) plot showing first (PC1) and second (PC2) principal components of a total of 11 cells from FACS sorted BRx68 morphometric subgroups based on their gene expression profiles. **B)** Bar graphs showing the top 10 enriched terms in Small vs. Large cells (n=781 genes) from the Reactome 2016 (signaling pathways), GO Biological Processes 2021 (cell processes/states) and the PanglaoDB Augmented 2021 (Cell types) databases. Enrichment analyses was performed using Enrichr based on upregulated genes. Colored bars correspond to terms with significant p-values (<0.05). An asterisk (*) next to a p-value indicates the term also has a significant adjusted p-value (<0.05).

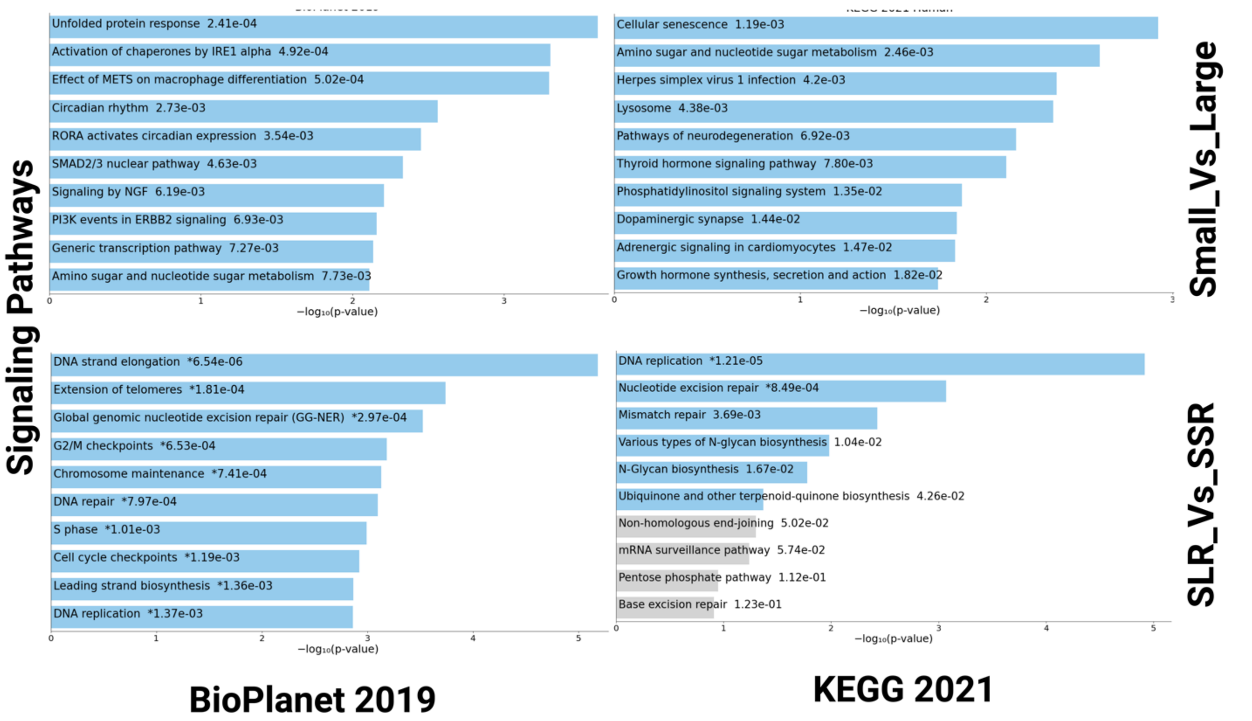

Supplementary Figure 4

Suppl Figure S4: Signaling pathways enrichment analyses of CTCs phenotypes. Bar graphs showing the top 10 enriched signaling pathways in Small vs. Large cells (n=781 genes) and SLR vs. SSR cells (n=79 genes) from the BioPlanet 2019 and KEGG 2021 libraries. Enrichment analyses were performed using Enrichr based on upregulated genes. Colored bars correspond to terms with significant p-values (<0.05). An asterisk (*) next to a p-value indicates the term also has a significant adjusted p-value (<0.05).
